# Using Mid-Upper Arm Circumference z-score (MUACz) tapes for community-based assessment and monitoring of nutrition risks among young children: a qualitative analysis of experiences and lessons from southwest Kenya

**DOI:** 10.1186/s12889-025-26095-5

**Published:** 2026-01-14

**Authors:** Dominic Bassah, Emilie McClintic, Ina Danquah, Amy R. Sharn, Suela Sulo, Erick Agure, Erick M. O. Muok, Grace Wothaya Kihagi, Raissa Sorgho

**Affiliations:** 1 Center for Wellness and Nutrition, Public Health Institute, Sacramento, CA USA; 2 Transdisciplinary Research Area “Technology and Innovation for Sustainable Futures” and Center for Development Research (ZEF), Rheinische Friedrich-Wilhelms University of Bonn, Bonn, Germany; 3 Heidelberg Institute for Global Health, University of Heidelberg, Heidelberg, Germany; 4 Global Medical Affairs and Research, Abbott Nutrition, Columbus, OH USA; 5 Global Medical Affairs and Research, Abbott Nutrition, Chicago, IL USA; 6 Kenya Medical Research Institute (KEMRI), Centre for Global Health Research (CGHR), Kisumu, Kenya

**Keywords:** Child malnutrition, Nutrition screening, Mid-upper arm circumference z-score (MUACz), Training, Community health, Nutrition indicator

## Abstract

**Objectives and study:**

In 2023, Community Health Volunteers (CHVs) and the Ministry of Health (MOH) staff were trained to use the novel MUAC z-score (MUACz) tape for household anthropometric measurements in southwestern Kenya as part of a randomized controlled trial titled ALIMUS-We are Feeding! In this qualitative study, we aimed to understand the experiences and lessons learned from using the MUACz tape for screening and monitoring among health professionals.

**Methods:**

In this qualitative study, Focus Group Discussions (FGDs) were used to explore the experiences of CHVs and MOH staff, who were trained on using the MUACz tape and applied the device for nutrition screening among 617 children aged between 23 and 59 months. Two FGDs were conducted with CHVs (*n* = 14) and one with MOH staff (*n* = 7). Trained facilitators led discussions using a semi-structured interview guide in Dholuo and Swahili. FGDs were recorded, transcribed, translated, and analyzed using thematic analysis.

**Results:**

Broad emerging themes showed that intensive training is essential for learning to use the MUACz tape. Trainings should be in an open learning environment, using simple language and incorporating hands-on practice. Participants had positive experiences using the MUACz tape, appreciating the expanded age, risk and color-coding categories. Challenges in using the tape included durability and the font size of tape markings. Participants emphasized the importance of integrating MUACz tape in existing CHV tool kits and data collection, in addition to adding z-score as an indicator in the health care database and services to ensure consistency and sustainability.

**Conclusions:**

The findings highlight that a few improvements of MUACz tapes could facilitate community-based nutrition screening and monitoring. Frontline community health workers play a critical role in shaping the implementation of public health programs. Accessible training is important for their buy-in.

**Supplementary Information:**

The online version contains supplementary material available at 10.1186/s12889-025-26095-5.

## Introduction

Severe acute undernutrition affects 19 million children globally and accounts for approximately 400,000 child deaths yearly [1]. In Sub-Saharan Africa, 32.3% of children under five are affected by stunting, 5.9% by wasting, and 3.8% by overweight. The 2025 edition of the Joint Child Malnutrition Estimates indicates that in East Africa, stunting affects 31.2% and wasting affects 4.8%. Approximately 3.9% of children in the region are impacted by overweight [2]. According to the 2022 Kenya Demographic and Health Survey (KDHS), stunting, wasting and overweight affected 18%, 5% and 3% of children under five, respectively [3].

Early detection and timely management are essential to prevent and treat malnutrition, especially in low- and middle-income countries [4]. One low-cost tool that has been effective in identifying undernutrition in conflict and low-resource settings is the Mid-Upper Arm Circumference (MUAC) tape. MUAC tape screens for severe acute undernutrition in children under five years of age, using a 3-color system signaling: red for severe undernutrition, yellow for moderate undernutrition, and green for well-nourished. Recommended by the World Health Organization (WHO) for use in communities, the MUAC tape has been instrumental in managing undernutrition in resource-restricted settings for over half a century [5, 6, 7] and has been used by both health care professionals (HCPs) and non-health care professionals(non-HCPs) [8–11]. Among HCPs, MUAC can be used as a single indicator for pediatric malnutrition. It has been validated to screen for malnutrition risk [12]. MUAC relies on fixed thresholds of 11.5 and 12.5 cm for defining malnutrition,it is limited to children aged 6 months to 5 years, does not account for child growth, and only identifies children with severe undernutrition. This leaves a significant number of unidentified children with malnutrition who may benefit from nutrition intervention.

**Fig. 1:**
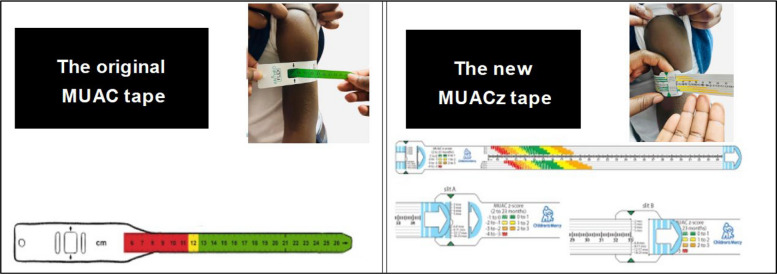
Original MUAC Tape and Updated MUACz Tape

In 2019, a new MUAC z-score (MUACz) tape was created as an innovation from the original MUAC tape to close this gap. The MUACz tape screens for over and undernutrition and expands on the utility of the MUAC tape by including an age range from 2 months to 17 years of age. In addition, it provides age-specific z-scores without the need to plot on a growth chart. The MUACz tape was developed by investigators at Children’s Mercy Hospital (Kansas City, Missouri, USA) to include age- and gender-specific color-coded reference data (i.e., z-scores) on the measurement tape, offering the added functionality of providing z-scores necessary for nutritional status monitoring without the need for reference charts or computations. The MUACz tape expanded age range means screening can be conducted in children and adolescents up to 17 years of age [13] (Fig. 1).

The tool has been used for the malnutrition screening of adolescents in nine countries across Europe, Pacific Asia, Africa, Latin America and North America in a non-healthcare setting [7]. In 2019, a study in Guatemala found that CHVs successfully used the MUACz tape to identify children and adolescents with malnutrition when provided with in-depth training and education [14]. A 2023 scoping review highlighted that the MUACz tape could be used for large-scale estimation of nutritional status and is a “favorable tool for easier and earlier diagnosis of pediatric malnutrition” among children and adolescents with or without intellectual and developmental disabilities [15]. 

In 2023, the ALIMUS – We are feeding! randomized controlled trial (RCT), which aims to evaluate the effects of home gardening and nutrition counseling on child nutrition, integrated the MUACz tape in the monitoring of nutrition status [16]. As part of assessing the effect of the intervention on malnutrition among the RCT study participants, CHVs and MOH staff were trained to use the MUACz tape as a nutrition screening tool.

This study sought to explore the experiences of Community Health Volunteers (CHVs) and Ministry of Health (MOH) staff on the use of MUACz tape as an anthropometric screening tool within the context of the ALIMUS RCT, recognized by Nature Medicine as a clinical trial that will change medicine in 2025 [17]. The specific objectives of this qualitative study were 1) to describe MUACz tape training experiences and preferences of CHVs and MOH staff, 2) to determine the acceptability of the MUACz tape according to CHVs and MOH staff experiences, and 3) to capture the recommendations of CHVs and MOH staff with regards to scaling up MUACz screening in Kenya.

## Methods

### Study design

We conducted a qualitative study using focus group discussions (FGDs) with MOH staff and CHVs. As part of the ALIMUS – We are Feeding! [16] RCT, MOH staff and CHVs in rural Siaya County in southwestern Kenya were introduced to and trained to use the MUACz tape for anthropometric screening. During their regular nutrition assessment visits to both control and intervention households, they employed the MUACz tape as a screening tool for the anthropometric measurements among children aged 23 to 59 months.

### Study participants and recruitment

This qualitative study was conducted in Siaya County, Kenya (population 1 million) within the Kisumu/Siaya Health and Demographic Surveillance System (HDSS) [3]. The RCT participants were drawn from the villages of Asembo, Gem and Karemo within Siaya County, where the majority speak Dholuo and practice subsistence smallholder farming, complemented by fishing activities. This contributes 70–90% to the livelihoods in this area [3].

The CHVs and MOH staff in our qualitative study have worked with the households of the ALIMUS RCT since 2021. They worked with 659 households with children under the age of five and their primary guardians, referred to as caregivers in the ALIMUS RCT.

As part of the integration of the MUACz tape into the nutrition screening in the ALIMUS RCT, the research team conducted training sessions with CHVs and MOH staff volunteers in Siaya. The trainings were conducted by co-author GK (researcher), OK (Siaya County nutrition coordinator), and JO (Siaya County field supervisor) in March 2023. All trainers are Kenyans with lived experience and understanding of the sociocultural landscape of the community. Fifteen hours of in-person training in groups were conducted for CHVs and MOH staff across three days, employing presentations, videos, and hands-on practice of anthropometric measurements using the MUACz tape. The MUACz training introduced the tape to the attendees, emphasizing its distinct features and sensitivity to age-specific malnutrition. The training equipped CHVs and MOH staff with the skills and hands-on experience to conduct nutrition screening using the MUACz tape. After learning how to use the MUACz tape, CHVs and MOH staff conducted screening for 617 children aged 23 to 59 months who were part of the ALIMUS RCT at 9 time points between April 2023 and March 2024. Household nutrition screening was conducted by CHVs using the MUACz tapes. Following the MUACz referral protocol, children with malnutrition risk were referred to the nearest health facility in the research area for further assessment and treatment. At the health facilities, health officers assessed malnutrition using MUACz tapes, anthropometry and assessment of clinical signs. Malnutrition was managed at the health facilities and follow-up was conducted by nutrition coordinators (MOH staff) who directly supervise the CHVs.

Purposive sampling was used to recruit a subsample from all MOH staff and CHVs involved in the ALIMUS RCT. Inclusion criteria comprised working in Siaya County, participating in the MUACz tape training, undertaking nutrition screening with the MUACz tape at two or more time points, and speaking English, Swahili, or Dholuo.

### Data collection

Focus group discussions (FGDs) were conducted by trained researchers from Kenya who were well-versed in English, Swahili, and Dholuo. A semi-structured interview guide was developed (Second section of Figure S1, S2) and pretested. The guide was tested first within the research team, then with CHVs from the ALIMUS project who received the MUACz training but were not part of the focus group discussions. The semi-structured guide was designed to allow for questions and answers to emerge naturally from field experiences and to encourage the study participants to take an active role in the discussion flow. Broad topics with key questions were used to guide the conversation. FGDs were recorded with an audio recording device, translated, and transcribed into English for analysis. The researchers assured participants of confidentiality during the consent process. FGDs were conducted in enclosed MOH halls chosen by the study team in collaboration with the County Government of Siaya. Study participants were assigned pseudo names (numbers) that they randomly picked from a box containing numbers generated by the study team commensurate with the total number of participants. Each participant mounted their number where they were seated. Participants were called by their number whenever they spoke. The same numbers were used in documenting the views and comments of the participants during the discussions. To promote open and productive discussions, FGDs were organized in separate cohorts-one consisting solely of CHVs and another of MOH staff. FGDs lasted between 72 and 110 min per session.

Furthermore, our team worked to mitigate the risk of losing information through translation by working with interviewers who spoke all three local languages: English, Swahili and Dholuo (EA, GK). Both the FGD interviewers were Kenyans with knowledge and understanding of the ALIMUS RCT and the qualitative study along with local and regional customs important for contextualizing findings. During transcription, the team engaged a translator with over 7 years of experience working across the three languages. Following each focus group, daily debrief forms (S3) were completed and weekly debrief meetings were held amongst team members along with memo writing by the principal investigator. The debriefing and memo writing were used to evaluate the progress of the work, review completed interviews and reflect on available data. Through this process, the principal investigator was able to determine data saturation.

### Data analysis

The FGDs were analyzed using thematic analysis. Data were coded inductively by the data analysis team and coding schemes were documented along with their central meaning, changes, and examples showing the code parameters. As this was an exploratory study, using inductive analysis was most appropriate to allow natural emergence of themes and highlight important nuances. Dedoose (version 9.2.12) software was used to organize, store, and visualize data. After coding, the relevant data was sorted into categories, further developed into themes, and then linked to overarching concepts. Results are presented following COREQ (S4) guidelines [18] on presenting and evaluating qualitative research. Data analysis included the triangulation of themes generated and field notes documented using the daily debrief forms by the research team. The same transcript was reviewed and coded independently by two team members, who met to review codes, labels, definitions and meanings. Upon merging of their codebooks, the two coders then proceeded to use this as a basis to code all the transcripts, including re-coding of the very first transcript. The coders then met to discuss the emerging points of interest in the data along the lines of the objectives of the study. Upon the first full draft of the study results, the authors conducted an in-person dissemination workshop in Kenya in February 2025. During the workshop, they presented preliminary results to the study participants who opted to participate (approximately half of those interviewed). Themes and key findings were presented for input, feedback and validation.

## Results

Three FGDs were conducted: two with CHVs (*n* = 14) and one with MOH staff (*n* = 7; Table 1). Quotes from the focus groups are labeled as follows for CHVs: “CHV1” and “CHV2”. The focus group with MOH Staff is coded “MOH”. Respondents are indicated using the letter “R” plus an assigned numeric code.

**Table 1:** Demographic information of study participants

**Study Cohort** **(FGD Code)**	**Participants, *n***	**Education Level**	**Age Range, *years***	**Work Experience** Range**, *years***
Community Health Volunteers (CHV1)	7	Primary (*n* = 2) Secondary (*n* = 4) College (*n* = 1)	41–61	14–21
Community Health Volunteers (CHV2)	7	Primary (*n* = 1) Secondary (n = 6)	36–60	5–31
Ministry of Health Staff (MOH)	7	Diploma (*n* = 3) Degree (*n* = 3) Higher diploma (*n* = 1)	35–50	1–19

### Intensive training is essential for CHVs and MOH staff learning to use the MUACz tape

CHVs (MUACz tape trainees) and the MOH staff (trainees, trainers and or observers of the training) described their learning experience of the MUACz training as engaging and conducive. FGD participants noted that this was because: 1) trainers created an open learning environment with a trusted relationship between researchers, CHV, and MOH staff; 2) trainers employed simple and understandable language during the training, facilitating understanding and comprehension; and 3) trainers presented the new information using well-prepared materials, practical application and hands-on learning (Table 2).

**Table 2:** Participant descriptions of essential factors for MUACz tape training

Code	Quote
Training occurred in an open and trusting learning environment	“*In the [training], I could see them [CHVs] acting very freely and [ALIMUS Researcher serving as MUACz tape trainer] could call them by their name and I saw them responding very happily… They also used to call [ALIMUS Researcher serving as MUACz tape trainer] by her name… and also [MOH Respondent NO.7] when they saw him, they would use his name…”* (MOH, R6)
Training with simple accessible language	*“What made it very easy for us was the trainer using the simplest language that we were understanding very first, they were also very friendly to us, you could ask questions and they didn’t get tired of you.”* (CHV2, R12)
Training with demonstrations and practical application	“*We enjoyed the training because we learned and after that, we went and applied it. There’s one day we found a child here (in the training venue) and after we used a video and now everyone practiced how to take readings from this child. This was good because one can see and practice it.”* (CHV1, R3)

### CHVs and MOH staff had overall positive experiences using MUACz tape

#### MUACz tape is user-friendly

In the FGDs post-screening, CHVs and MOH staff expressed an overall positive opinion of the MUACz tapes. CHVs described how several enhancements in the MUACz tape facilitated screening in comparison with the original MUAC tape. They appreciated details on the MUACz tape that are not present in the original MUAC tape, such as 1) new colors that correlated to new information on nutrition risk status; 2) the expanded age categories and nutrition risk status categories on the MUACz tape; 3) and the z-score measurement which facilitated assigning a nutritional risk status to children upon screening without the need for additional calculation or computation. Table 3 shows the participants’ description of the user-friendliness of the MUACz tape.

**Table 3:** Participant descriptions of the user-friendliness of MUACz tape

Code	Quote
Extended tape colors (green, yellow, orange, red) are useful and informative	“*[This MUACz tape] shows us all the colors according to how you taught us. It shows us if the child is okay, mild or malnourished. […] So, this is helpful. And the advantage is that it’s also available for adults.”* (CHV1, R2)
Inclusion of extended age categories, MUACz increased the ability to screen children	“*What made this easy was that the age category is ever there. When, I have a child who is 24 months, and his or her age is falling under 24 to 29 months, then I just do the measurement and read where the arrow is. And if it is at green dotted, I'll just go to the Z-score and know where the child is falling.”* (CHV1, R1)
Z-score directly on the tape facilitates the interpretation of nutrition risk status	“*The good thing with this [MUACz tape] is while using the MOH booklet when weighing and entering, I used to see minus two, minus three. I didn’t know about the z-score. All I knew was reading the red or yellow or when it’s 2 cm, 3 cm, 13 cm but I didn’t know the meaning of z-score… Now since I began using this one [MUACz tape], even when screening a child, I know how to read and interpret the measurements.”* (CHV1, R5)

Lastly, CHVs shared that after the initial apprehension of having a child examined, the caregivers displayed curiosity and interest in the MUACz tape “*…the women [in the household] were also very keen, they had gotten used to the old one [original MUAC tape], so when you were with the child, they were very keen to see what you were doing, they wanted to know how that thing [MUACz tape] worked and wanted to know how it was used on the child. They were very keen and very sensitive.”* (CHV2, R9).

#### MUACz tape durability poses challenges

While FGD participants expressed a positive experience and a favorable outlook on the MUACz tape utility, participants stated frustrations with the material and manufacturing attributes of the tapes including: 1) the small font size on the tape; 2) the fading color of the tape after 60–80 screenings in three months; and 3) the limited durability of the tape material (Table 4). These emerged as challenges in the use of the tape.

**Table 4:** Participants’ descriptions of challenges faced when using MUACz tape

Code	**Quote**
MUACz tape font size readability was a challenge	*“…they should just change the writing, should use a bigger font. The reason is, that when you are in the household, at times there is partial darkness. So, the numbers are not coming out clearly.”* (CHV1, R12)
MUACz tape color (intensity) fades over time with repeated use	“*We need to have color intensity. It disturbs me to [have the color] graduate from red to this other which is brown, and as time goes by it fades. I think because of the material”* (MOH, R7)
MUACz tape durability is limited (material tears and rips over time)	“*As I'm looking at the [MUACz] tape, it is doing well but it's not durable. It is not as durable as the [original] MUAC. If they can use the materials that were used on that one [original MUAC]it can last longer.”* (CHV1, R5)

### Integration of MUACz tape into existing health care services and systems is necessary for screening sustainability

FGD participants suggested approaches to integrating MUACz tape nutrition screening and screening data into routine household healthcare delivery and in the healthcare system of the MOH. The main suggestions outlined included: 1) expanding household and community awareness of nutrition and nutrition screening services 2) garnering MOH buy-in and closing gaps in nutrition screening knowledge amongst MOH ranks are essential to its consistent use in the community and 3) incorporating MUACz data as an indicator into existing local and national MOH databases.

### Nutrition services awareness is the first step to using MUACz in communities

FGD participants noted that increasing general community awareness around the importance of childhood nutrition would facilitate household-based nutrition service delivery. This will address one of the barriers faced by CHVs in conducting screening. CHVs also expressed the importance of targeting and involving household influencers to raise general community awareness and reduce the anxiety of caregivers.

Household influencers are described as decision-makers in the household with influence on a child’s nutrition, such as fathers/male caregivers, and in-laws/grandmothers. Participants emphasized the impact of household power dynamics on successful nutrition counseling and MUACz screening. Table 5 shows participants’ descriptions of household factors that influence screening acceptability.

**Table 5:** Participants’ descriptions of household factors that influence screening acceptability

Code	Quote
Awareness creation to reduce barriers to screening	*“it was like initiating a new thing to these people [caregivers] that they were not taught about. It was like they [caregivers] were just told they could be examined, and they didn’t know what equipment was to be used. By the time you tell the parents that you want to examine the child and know the amount of food in the body, the woman already thought it was an injection. Even if you use this [MUACz tape], they still think you want to inject the child.”* (CHV2, R14)
Household influencers impact successful screening	“*The grandmothers are also influencing the decision within the household. So, we go and counsel because counselling is one-on-one and then I just leave the house. So, the next time you see the mother-in-law, seeing you what you're doing, probably they do not approve of that.” (*MOH, R7)

### Garnering MOH buy-in and closing nutrition screening knowledge gap amongst MOH staff

Participants shared that integrating the MUACz tape into the existing health care and health delivery system requires: 1) MOH buy-in from the start; 2) training to address and close knowledge gaps around the MUACz tape; 3) availability and access to the MUACz tape through its addition to CHVs toolkits; and 4) the routine collection of MUACz tape data at local level which feeds into national nutrition data reporting. To integrate MUACz screening into the health system, participants recommended addressing knowledge gaps by expanding MUACz training to include supervisors at the MOH, CHAs, and other members of the community healthcare team who did not receive training by ALIMUS RCT staff on nutrition counseling or screening (Table 6).

**Table 6:** Participants’ descriptions of integrating MUACz into the MOH routine processes as essential to its use in community setting

Code	Quote
Expanded MUACz training	“*Training should go to the CHVs, all the CHAs, all the nutritionists, and all our healthcare workers who deal with infants, and more so, we should have a representative in each facility. So that once we have such children, there's room for counseling on this topic or nutrition. So that if they miss it at the community intervention, they can still get it at the facility level.”* (CHV2, R14)
Buy-in and ownership of MUACz screening program	“*We [MOH staff] need to own it, we need to give it to facility staff, and CHVs so that they own it […] I've heard them say ‘that they are using [MUACz tape as] research gadgets, MUACz [tape] for research not knowing that it's a magic bullet.”* (MOH, R6)
Access to MUACz tapes	*“… This means two ways support us to proceed, avail, and train them on the use [of MUACz tape]*…* “that will allow the second bit which is putting this [MUACz tape] into the CHVs kit. So that just flows. It will generate so much nutrition data.”*(MOH, R7)

Participants indicated that CHVs’ access to the MUACz tape was a factor that could influence the successful integration of MUACz screening into routine community-level anthropometric measurement. In 2024, the government of Kenya formalized the role of CHVs in the Kenyan healthcare system and equipped them with “community health kits”. CHVs and MOH staff in our study expressed that the MUACz tape should be made more readily available and accessible to all healthcare professionals along the path to care continuum from detection to treatment and expressed that the addition of the MUACz tape to these “kits” following training would be instrumental. They emphasized the importance of securing “buy-in” of the MUACz tape across ministry levels, including the MOH, facility-level staff (Community Health Assistants (CHAs) who oversee CHVs, nutritionists…), and CHVs for successful nutrition screening programs beyond the ALIMUS RCT.

### Incorporating MUACz data as an indicator into existing local and national MOH databases can facilitate evidence-based decision making

Participants shared that systematic recording of nutrition screening results from MUACz tape will facilitate evidence-based decision making locally. They also noted that integrating space for MUACz indicators in local and national nutrition databases and MOH reporting tools will facilitate MUACz tapes' use within the existing healthcare systems, especially in rural communities. As illustrated in Table 7, participants indicated that the addition of MUACz tapes to the CHV community care toolkits will enable consistent data collection, reporting and integration into local and national public health databases and thus provide more data for the MOH to consult for decision making.

**Table 7:** Participants’ descriptions of incorporating MUACz data as a key indicator into existing MOH local and national databases

Code	Quote
Integration of MUACz data into reporting tools	“*…integrating the thresholds into the reporting tool, the routine reporting tool, but again that is done at the national, so that we capture the z-score and the MUACz [tape] reading. Right now, it's the only [original] MUAC reading in the official reporting tools.”* (MOH, R7)
Addition of MUACz to the CHV toolkits for community work to improve data capture	*“…adding in the CHV kit, [means] having it in for our reporting because currently … [we] don’t have a reporting tool that captures the details”* (MOH, R6)
Incorporation of MUACz into national guidance	*“the IMAM (Integrated Management of Acute Malnutrition) guidelines are being reviewed. But it has not incorporated the new MUAC […] if it is incorporated into that [the IMAM], maybe this way forward screening identification for malnutrition would be so easy”. (MOH, R7)*

## Discussion

### Summary of findings

**Fig. 2:**
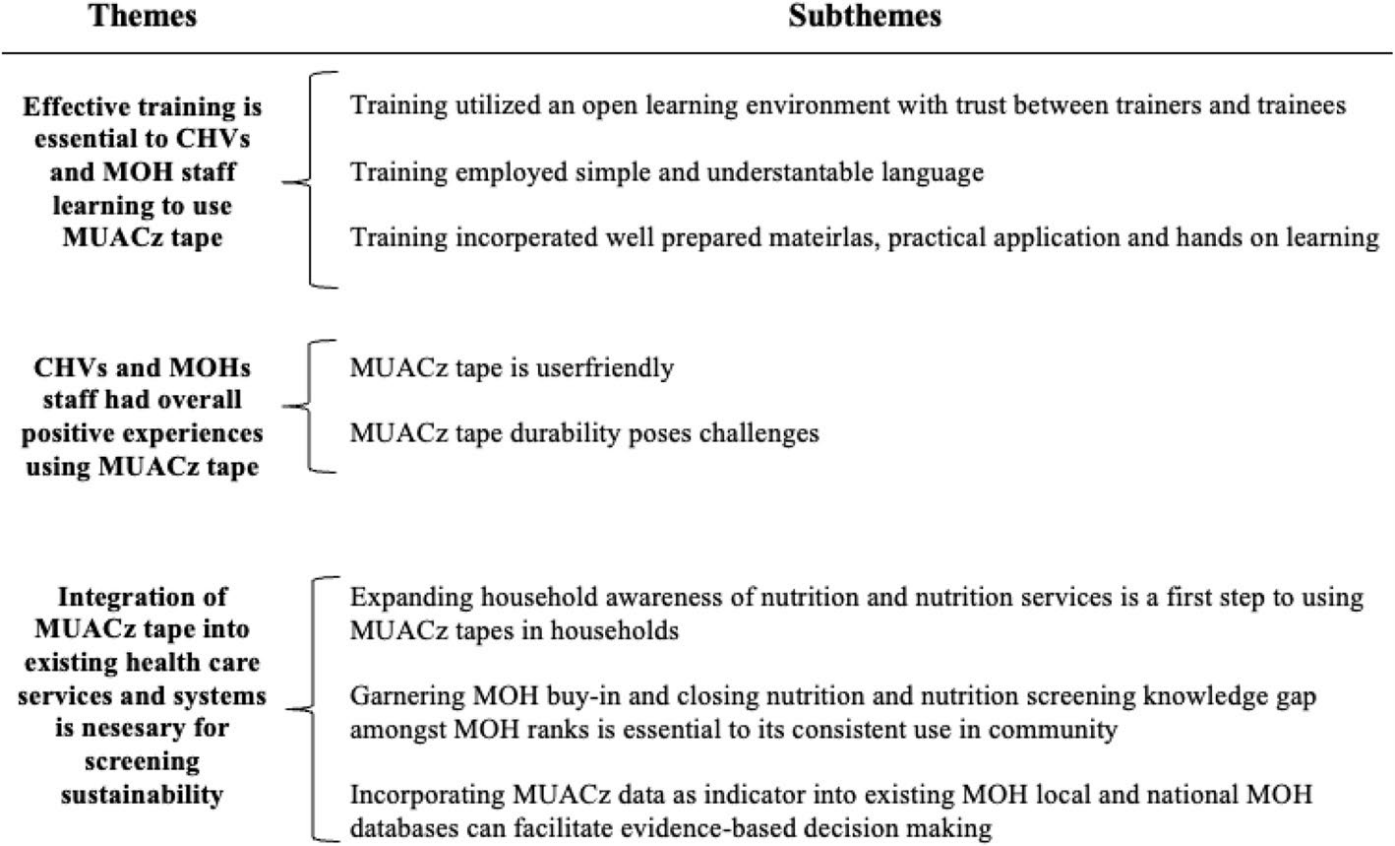
Overview of the themes and categories that emerged from the focus group discussions with Community Health Volunteers (CHVs) and Ministry of Health (MOH) staff on the training, implementation and scaling of Mid-Upper Arm Circumference z-score (MUACz) tape screening in communities

Integration of the MUACz tape into the ALIMUS RCT was feasible because i) the MOH staff and CHVs received thorough training on the MUACz tape, ii) facilitators developed strong rapport with participants, iii) the learning environment was open and interactive, iv) the language and the content of the hands-on training were understandable. Participants expressed that the MUACz tape allowed for easy interpretation and use while screening for malnutrition through color-coding and expanded age categories, among communities in Siaya County. Participants shared that the durability of the MUACz tape was relatively poor due to structural and color deterioration over time. To integrate the MUACz tape into the existing health system, participants recommended developing household and community awareness by connecting with community and household influencers, ensuring MOH ownership of the project by providing training and MUACz tapes, and integrating systematic MUACz data collection and input into the existing local and national health programs and data systems. Overview of the themes and categories that emerged from the focus group discussions is presented in Fig. 2.

### MUACz tape for nutrition screening is feasible

Anthropometric nutrition screening tools are essential for early detection and treatment of malnutrition, as well as for ongoing community surveillance and public health planning efforts. Though the original MUAC tape is commonly used for nutrition screening, the MUACz tapes may be an impactful alternative in community settings when z-score diagnostic resources are limited. Our findings among MOH staff and CHVs with MUACz tape use for household nutrition screening are consistent with the literature that caregivers and community health workers have the capacity to use MUAC [19, 20] for the detection of severe acute malnutrition after a short training [21]. As in our study, effective training has been highlighted in literature as essential in scaling up household MUACz tape screening [7]. The feasibility of the MUACz tape screening may also be attributed to the tool’s simplicity and reliability, and the ability of nonprofessionals to use the tool after minimal training as observed by Sharn et al. [7] in their study involving the use of the MUACz tape by non-healthcare personnel in children and adolescent sports programs. The acceptance of the MUACz tape as a screening tool for household-level anthropometry can be attributed to the non-invasive nature of the tape and its similarity to the traditional MUAC tape which is widely accepted in community settings. To further increase acceptability and facilitate a smooth nutrition screening, participants in the study suggested increasing community-level awareness creation and sensitization taking into consideration dynamics in household decision-making to include household members other than parents of the children in conversations surrounding childhood nutrition.

### Integration of MUACz into the health system as a screening tool

FGD participants emphasized data collection system integration as a preferred approach for rolling out the MUACz tape for screenings in the rural context. They expressed a need to integrate the MUACz tape measures into the national MOH reporting tool and provision of training to staff to ensure efficient roll-out of MUACz screening, reporting, and integration into routine anthropometric measurements. As indicated by participants, the successful integration and scaling up of MUACz screening is contingent on buy-in from implementers, improvement in the durability of the tape and adequate training of frontline health professionals and community volunteers.

Various innovations in anthropometric screening tools for diagnosing undernutrition have emerged over the years, including the CIMDER tape, the Click-MUAC, and the uniMUAC tape. The CIMDER tape includes two tapes – one for female children and one for male children – and has cut-off points for distinct age groups (3–6; 6.1–18; 18.1–36 and 36.1–60 months). However, this tape has not been scaled into health systems or services, is limited to children under the age of 5, and has mainly been used in research studies [22, 23]. The Click MUAC and uniMUAC have been used in conflict zones and humanitarian settings or in pilot studies, whereby CHVs have been trained to use the tape. Similarly, the Click-MUAC and the uniMUAC are not integrated into existing health and data systems and services [24, 25]. Salam et al. [26] stated that the absence of nutrition indicators in the existing health information system is a barrier to integrating health programs into local and national health care systems. This is in alignment with recommendations from our study to include nutrition indicators such as MUACz in systematic data collection and reporting at both community and clinic levels. The direct inclusion in local and national data management systems ensures nutrition status is measured, recorded, and analyzed to facilitate the design of locally relevant interventions and equitable distribution of resources. Integration of MUACz tape screenings into the health system and services requires stakeholder awareness, coordination, effective logistics systems, and existing facility and community-level staff with adequate training to implement MUACz tape screening as concluded by Bilah et al. [27]. The need for training was highlighted in the acknowledgment of a knowledge gap between CHVs (the implementors) and CHAs (the supervisors). FGD participants unanimously agreed that the knowledge gap of CHAs and other health care personnel along the nutrition path to care in Siaya must be addressed. They suggested this could be done with consistent training across the relevant spectrum of health systems. Owing to the user friendliness of the MUACz tape, screening with the new tool could be feasibly incorporated into the Integrated Management of Acute Malnutrition guidelines in Kenya to encourage regular standardized screening and reporting of malnutrition, contributing to national efforts towards the reduction in childhood malnutrition.

### Strengths and limitations

The ALIMUS RCT, prior to its use of the MUACz tape, worked closely for three years with the Siaya County MOH and the five communities where the MUACz tape screening was conducted. The trust-built working relationship between the project, MOH, and community facilitated the integration of the tool into the ongoing work, with the full support of the Siaya County MOH and without disruption of routine health and nutrition activities. Our study’s inclusion of both CHVs and MOH staff provided the opportunity for a contextual assessment of the project across different sectors of the health delivery spectrum. Participants had ample time for onsite use of the MUACz tape and were therefore in the best position to share their experiences and recommendations on improving the design, application, and implementation of the tool. The study also benefited from expertise from the Kenya Medical Research Institute (KEMRI), the premier national body responsible for health research in Kenya. Our study used the Consolidated Criteria for Reporting Qualitative Research (COREQ) [18] to ensure all essential aspects of the work were communicated in our results to increase the transferability of these results to similar contexts and settings.

A limitation of this study is the translation of raw data that is inherent to all qualitative work completed and disseminated in different languages, where loss of meaning can occur. This risk was mitigated by the cumulative expertise and experience of the research team in Kenya.

### Perspectives for research and policy

While the MUACz tape was perceived as feasible to use by MOH staff and CHVs in the ALIMUS RCT study, improvements around the durability of the tape were recommended prior to sustained use and scalability. Larger feasibility and validation studies with the MUACz tape should be conducted among low-resource communities where the MUACz tape is poised to make the most impact. Though our study focused on community use, research and evidence within clinical settings (health posts, clinics, hospitals) would provide additional essential information on the adoption of the MUACz tape by health professionals and care providers across the health care system. The promotion of the use of MUACz tape for the screening of children and integration of the data into the existing health databases presents the opportunity for evidence-based policies and interventions aimed at preventing and reducing childhood malnutrition. Systematic MUACz data collection, analysis and sharing with stakeholders can provide opportunities to assess the impact of MUACz screening towards national and local level nutrition goals and objectives. The capability of the MUACz tape to provide an age-specific z-score in a one-step process makes it a viable screening tool for the implementation of strategies and activities aimed at achieving key results areas on the Prevention and Integrated Management of Acute Malnutrition (IMAM) as stated in the Kenya Nutrition Action Plan (KNAP) 2018–2022 [28]. MUACz screening can be integrated into interventions aimed at expanding nutrition screening at the community level and improving early detection, treatment and monitoring of children affected by malnutrition. Knowledge and the skills of using the MUACz tapes for screening can contribute to capacity building, leading to expansion of screening and the integration of IMAM services with other programs in the continuum of care for children.

## Conclusion

Our findings indicate that the MUACz tape can be used by community health workers in low-resource settings to conduct community-based nutrition screening of children. The study underscores the critical role of frontline community health workers in shaping the implementation of public health programs, the importance of obtaining community buy-in, and the necessity of providing proper training before community-level program implementation. Lastly, our study suggests that the scale-up and out of MUACz tape use in routine local and national nutrition screening and monitoring programs may be possible if sustainably integrated into existing health management systems and supporting the design of locally relevant interventions.

## Supplementary Information

Supplementary Material 1.

Supplementary Material 2.

Supplementary Material 3.

Supplementary Material 4.

## Data Availability

The datasets generated and/or analyzed during the current study are not publicly available to preserve participant privacy but are available from the corresponding author on reasonable request.

## Abbreviations

ZEF: Center for Development Research
KEMRI: Kenya Medical Research Institute
CGHR: Centre for Global Health Research
CHV: Community Health Volunteer
MOH: Ministry of Health
MUAC: Mid-Upper Arm Circumference
MUACz: Mid-Upper Arm Circumference z-score
FGD: Focus Group Discussion
WHO: World Health Organization
KDHS: Kenya Demographic and Health Survey
HCP: Health Care Professionals
RCT: Randomized Controlled Trial
HDSS: Health and Demographic Surveillance System
COREQ: Consolidated Criteria for Reporting Qualitative Research
CHA: Community Health Assistant
IMAM: Integrated Management of Acute Malnutrition
KNAP: Kenya Nutrition Action Plan
WMA: World Medical Association
SERU: Scientific and Ethics Review Unit
DFG: German Research Foundation
