# Supplementary material for: Using Mid-Upper Arm Circumference z-score (MUACz) tapes for community-based assessment and monitoring of nutrition risks among young children: a qualitative analysis of experiences and lessons from southwest Kenya
Source: BMC Public Health. 2026 Jan 14;26:351. doi: 10.1186/s12889-025-26095-5 (PMC12849407; doi:10.1186/s12889-025-26095-5)
Supplement: Supplementary file 1 — Supplementary Material 1. [file 12889_2025_26095_MOESM1_ESM.pdf]

ALIMUS: *Home gardening and nutrition counselling in rural Kenya: A qualitative study on knowledge gain*

Interviewer: Complete these steps before starting the interview

|__| Introduce yourself to the participant (CHV)
|__| Ensure you are with the right participant in a place he/she can speak uninterrupted and freely
|__| Ensure the participant reads and understands the "Information Form" and the "Consent Form"
|__| Fill out and sign the "Consent Form" (2 copies, one for the interviewer & one for the participant)
|__| Fill out the "Participant Identification Form"
|__| Turn on your audio recording device to start the interview.

Interviewer: Introduce the structure of the planned interview. State that you will ask some general questions and that you then move to the experiences of the participant with home gardening and/or nutrition counselling.

Introduction

1. Please introduce yourself and tell us about your role in the project.
2. Did you have experience with Nutrition Counseling implementation **before** this project?

Nutrition Counseling

3. How has your experience with nutrition counseling implementation been this far in the project?
4. What **topics/subjects/ concepts** have you learned while implementing NC intervention?
5. Which **topics/subjects/concepts** were **entirely new** to you?
6. Which topics/subjects were **most valuable** for you to learn about? (why)
7. How have you applied/planned to apply the new information/knowledge gained in your life/career?
8. What **was easy** and what was **difficult** about implementing the NC sessions/project?
9. What recommendations would you give for improving/continuing NC projects in future?

MUAC screening and implementation

10. Do you have prior experience using a MUAC tape for nutrition screening?
11. What are your thoughts about the new MUACZ tool?
12. What are your recommendations for changing/adapting/improving the MUACZ tool?
13. What was **easy** and what was **difficult** about **learning** the MUACZ tool?
14. What was **easy** and what was **difficult** about **using the** MUACZ tool on children?
15. What systems and support (training, materials …) do you need to continue implementing nutrition screening with the MUACZ tool?
16. What challenges do you foresee in scaling up MUACZ across the region? (Scaling to other households, communities, across the county and the country)

Closing questions

17. What are your final thoughts/lessons learnt about nutrition counselling intervention?
18. What are your last thoughts on the MUACZ tape?

Interviewer: Thank the participant for their time and efforts
