# Supplementary material for: Using Mid-Upper Arm Circumference z-score (MUACz) tapes for community-based assessment and monitoring of nutrition risks among young children: a qualitative analysis of experiences and lessons from southwest Kenya
Source: BMC Public Health. 2026 Jan 14;26:351. doi: 10.1186/s12889-025-26095-5 (PMC12849407; doi:10.1186/s12889-025-26095-5)
Supplement: Supplementary file 3 — Supplementary Material 3. [file 12889_2025_26095_MOESM3_ESM.pdf]

ALIMUS: *Home gardening and nutrition counselling in rural Kenya: A qualitative study on knowledge gain*

Post Interview Daily Debriefing Form

Name of Interviewer(s) :______________________ Date:____/_____/____

Study site:___________________ Study ID of interviews: ____________________ Interview type: FGD/IDI

	Question/Theme	Note / Response
Q01	What have you heard/observed today that has **caught your attention**?<br><br>Why? Who said it?	
Q02	What have you heard/observed today that you thought was **most important**?<br><br>Why? Who said it?	
Q03	What insight into the **experience** of households / stakeholders on the HG & NC projects have you gained today?	
Q04	What insight into what households / stakeholders have learnt of **home gardening Intervention** have you gained today?	
Q05	What insight into what households / stakeholders have learnt of **nutrition counselling intervention(including use of MUAC z-scores)** have you gained today?	
Q06	Has anything you heard/seen today **surprised you**?<br><br>Why? How?	
Q07	Is there any **sentence you heard today that sticks in your mind**? In what context did it emerge?	
Q08	Are there any **thoughts/emotions/experiences that keep reappearing** throughout data collection?	
Q09	**Any other thoughts** from the day that you would like to share?	
